# Research priorities for diagnostics, progression monitoring, and treatment of glaucoma in myopic eyes

**DOI:** 10.1038/s41433-026-04549-3

**Published:** 2026-05-21

**Authors:** Sanna Leinonen, Rupert Bourne, Adam LaMontagne, Gordana Sunaric Mégevand

**Affiliations:** 1 Tays Eye Centre, Tampere University Hospital, Tampere, Finland; 2 Faculty of Medicine and Health Technology, Tampere University, Tampere, Finland; 3 Cambridge University Hospital, Cambridge, UK; 4 RWS Language Weaver, Maidenhead, UK; 5 Centre Ophtalmologique de Florissant, Fondation A. de Rothschild, Geneva, Switzerland

**Keywords:** Outcomes research, Optic nerve diseases

## Abstract

**Objective:**

The aim of this study was to report results of a survey on the diagnostics, progression monitoring, and treatment of glaucoma in myopic eyes and identify future research priorities.

**Methods:**

We conducted a survey among the members of the European Glaucoma Society to explore their perspectives on the most critical questions regarding glaucoma in myopic eyes. We asked participants to compose critical questions about glaucoma diagnostics, progression monitoring, nonsurgical, and surgical management in myopic eyes. We used large language models to identify the most frequently recurring themes in the questions and human evaluation to validate and calculate responses for each theme.

**Results:**

A total of 251 participants out of 1045 responded, of whom 98% were glaucoma specialists, and 86% glaucoma surgeons. We received 581 questions on diagnostics, 470 on progression monitoring, 453 on nonsurgical treatment, and 483 on surgical treatment of glaucoma in myopic eyes. The top themes for diagnostics and progression monitoring were differentiating glaucomatous changes and progression from myopic changes and progression, role and reliability of ocular coherence tomography, and visual field testing and interpretation in myopic eyes. For treatment, the leading themes were choice of surgical procedure, effectiveness of different medications, and effectiveness of selective laser trabeculoplasty in the treatment of glaucoma in myopic eyes.

**Conclusions:**

The survey showed that there are knowledge gaps regarding diagnostics, monitoring, and treatment of glaucoma in myopic eyes among glaucoma experts. We recommend that the most frequently recurring themes of this survey are prioritised in future research in this area.

## Introduction

Myopia is a growing world-wide health problem. It was estimated that in 2000, 23% of the world population was myopic, with a refractive error of -0.50D or less and 3% had myopia with a refractive error of -5.0D or worse. The prediction is that by 2050, half of the world population will be myopic, and 10% will present with high myopia [1]. Primary open-angle glaucoma, normal tension glaucoma, and less commonly, pigmentary glaucoma and primary angle-closure glaucoma have been reported in myopic eyes [2–5]. Increasing axial length is associated with an increased risk for primary open-angle glaucoma (POAG) [6–13], with a pooled odds ratio of 1.8–1.9 for POAG [14, 15].

Myopia, and especially high myopia, can be associated with clinical features that resemble glaucomatous damage, which poses challenges in the diagnosis and monitoring of glaucoma. In clinical practice, it is challenging to distinguish myopic structural and functional changes from glaucomatous findings [16–21]. Furthermore, glaucoma treatment, either surgical or nonsurgical, has not been studied sufficiently in eyes with high myopia [22–33].

We recognise that an effective research system should target topics such as interventions and outcomes that clinicians consider relevant, without ignoring the need to improve and replicate existing research within the same topic areas [34]. In 2024, we conducted a survey among the members of the European Glaucoma Society to explore their perspectives on the most critical questions in glaucoma diagnostics, progression monitoring, nonsurgical, and surgical treatment in myopic eyes. For this study, we analysed the survey results to identify future research priorities in the field of glaucoma and myopia.

## Methods

This survey was the first step in an ongoing international Glaucoma and Myopia Project dealing with glaucoma in myopic eyes. The survey was designed to collect and summarise the most critical and challenging questions in diagnostics, progression monitoring, nonsurgical, and surgical treatment of glaucoma in myopic eyes. All ordinary and extraordinary European Glaucoma Society members were invited by email to participate in the online survey. These ordinary and extraordinary members with a specific interest in glaucoma are ophthalmologists or researchers (PhD) who typically are glaucoma specialists. Ordinary members practice within Europe, while extraordinary members practice outside Europe and have a close relationship with the society's activities. The survey was open from November 19, 2024, to December 11, 2024.

We collected background information, including country of work, level of specialist expertise, and an estimate of how many myopic glaucoma suspects or myopic glaucoma patients seen by the participants per week. No personal information, such as name, email, age, or sex, was collected. As the survey was conducted anonymously, with no access to participant identifiers, formal ethical approval was not requested. Informed consent was implied by voluntary participation in the study.

We asked the participants to compose “the most challenging and critical questions” regarding glaucoma in myopic eyes. The task was divided into 4 categories, as follows: diagnostics, progression monitoring, nonsurgical treatment, and surgical treatment of glaucoma in myopic eyes. For each category, we requested the participants to compose 1–3 questions.

Background data and number of responses are reported as rates and numbers. To analyse the free text regarding the most critical questions, we used a combination of large language models (LLMs) Microsoft Copilot and ChatGPT [35, 36]. We prompted the LLMs to analyse the survey responses, to provide a list of the top ten themes that can be used as subcategories to categorise the responses, including the number of questions that fall into each category, and label the responses with theme names. We told the LLMs that the themes may or may not overlap between the categories. This prompt was given separately for each category of questions, for diagnostics, progression monitoring, nonsurgical treatment, and surgical treatment. SL confirmed the labelling and number of responses from the original survey data. If the number of responses did not match the original data, the LLMs were prompted to recalculate the answer. In case of disagreement, human judgement was preferred. To summarise our findings, we report the 10 most common themes in the survey as well as the number of questions in which the themes appear. We also report the most common themes for each survey category and 3 most common themes for combined categories: diagnostics and progression monitoring and surgical and nonsurgical treatment. To illustrate each theme, we hand-picked some of the most representative questions from the survey. The original questions are copied from the survey unedited, and they are written in italics.

## Results

We received responses from 251 of 1045 (24%) EGS members, of whom 82% worked in Europe. Most participants (98%) described themselves as glaucoma specialists, and 86% as glaucoma surgeons. The background characteristics of the participants are listed in Table 1.

**Table 1:** Background of the 251 survey participants.

Glaucoma specialist	98%
Glaucoma surgeon	86%
**Stage of expertise**	
Senior specialist	86%
Junior specialist	7%
Researcher, PhD	4%
Fellow	2%
Resident	1%
**Country of workplace**	
European country	82%
United Kingdom	13%
Turkey	8%
Spain	7%
Italy	6%
Greece	6%
Austria	5%
Other European country	37%
Extra-European country, each <5%	18%
How many myopic glaucoma suspects or myopic glaucoma patients do You see/treat per week on average?	
>20	13%
6-20	45%
1–5	40%
0	2%

### Most critical questions

We received 1987 responses for the most challenging and critical questions in the diagnostics (*n* = 581), progression monitoring (*n* = 470), nonsurgical treatment (*n* = 453), and surgical treatment (*n* = 483) of glaucoma in myopic eyes. Table 2 presents the 10 most common themes for the most critical questions to be answered regarding glaucoma in myopic eyes.

**Table 2:** The 10 most common themes in the survey for the most critical questions for glaucoma diagnostics, progression monitoring, nonsurgical, and surgical treatment in eyes with myopia.

Theme of the critical question		Number of questions
1	Differentiating glaucomatous changes / progression from myopic changes	209
2	Choice of surgical procedure	181
3	Role and reliability of OCT	172
4	Visual field testing and interpretation	135
5	Effectiveness of different medications	111
6	Progression analysis with different devices and programs	108
7	Effectiveness of SLT	106
8	Role and reliability of IOP measurement	91
9	Surgical risks	88
10	Target IOP	71

*OCT* optical coherence tomography, *IOP* intraocular pressure, *SLT* selective laser trabeculoplasty.

The most common theme in our survey was how to distinguish glaucomatous changes and glaucomatous progression from myopic changes and progression (209 questions). Representative questions included:

- *How to distinguish myopic changes on OCT and visual field from glaucoma changes?*
- *How can I/we be sure that the myopic disc is a glaucomatous one?*
- *Is this progressing functional loss due to progressing glaucoma or myopia?*
  Respondents discussed the role and reliability of ocular coherence tomography (OCT) in 172 questions, examples of which are as follows:
- *What is the role of OCT macula parameters in glaucoma diagnostics in myopic eyes?*
- *What is the value of OCT when the optic disc is enlarged, flattened and has deformations secondary to myopia?*
- *Which OCT parameter is best for measuring progression in high myopia (if any)?*
- *How reliable is follow-up with OCT in myopic patients?*
  Visual field testing and interpretation recurred as a theme in 135 questions:
- *Which are the typical visual field defects in high myopia and in glaucoma on a myopic patient?*
- *What is the preferred perimetric strategy to follow progression in myopic glaucoma?*
- *What is the normal fluctuation of visual field sensibility (dB) in myopia?*
  How to best analyse progression was the theme in 108 questions:
- *What should be the frequency of testing of a myopic glaucoma suspect?*
- *Would you rely more on OCT or more on VF in the follow-up of (high) myopic eyes with glaucoma?*
- *What is the first indicator that glaucoma is progressing in myopes?*
  Role and reliability of the intraocular pressure (IOP) measurement was asked 91 times in the survey, and there were an additional 71 questions regarding target IOP in the survey. These questions represent the most typical questions in the survey:
- *What is the role of IOP level in glaucoma diagnostics in myopic eyes?*
- *Can we fully rely on IOP or should we integrate corneal hysteresis into the equation?*
- *What is the role of IOP in glaucoma diagnostic in myopic eyes after refractive surgery?*
  The second most popular theme in this survey was choice of glaucoma surgery with 181 questions which are illustrated by these questions:
- *What is the surgery of choice as a primary procedure in high myopes?*
- *Which surgical treatments provide lowest pressure?*
- *What role do minimally invasive glaucoma surgeries (MIGS) play in treating myopic glaucoma, and are they equally effective?*
  Risks in surgical treatment of glaucoma in myopic eyes were considered in 88 questions, of which 35 addressed postoperative hypotony.
- *Which type of glaucoma surgery is the safest for myopic eyes?*
- *Does trabeculectomy have higher complication rate in myopic eyes?*
- *Are there any biomarkers to predict risk of early hypotony in trabeculectomy or shunt surgery?*
  Effectiveness of different IOP-lowering medications was the main theme in 111 questions, with an additional 47 questions mentioning different drug classes written as one-word responses. These questions cover the most common questions within this theme:
- *Are IOP lowering medications equally effective in myopic patients compared to non-myopic patients?*
- *Which IOP-lowering medication is the most effective in myopic eyes?*

Effectiveness of selective laser trabeculoplasty was addressed in 106 questions, the most common being:

- *Is SLT as effective as in POAG in myopic eyes?*
- *Should SLT be used as first choice?*

The remaining themes recurred fewer than 40 times in the survey, each theme in less than 2% of the questions. Table 3 presents the 5 most common themes, some of which overlap, for each category of the survey.

**Table 3:** The 5 most critical question themes in diagnostics, progression monitoring, nonsurgical treatment, and surgical treatment of glaucoma in myopic eyes.

Diagnostics		Number of questions
1	Differentiating glaucomatous changes from myopic changes	117
2	Role and reliability of OCT	115
3	Role and reliability of IOP measurement	65
4	Visual field testing and interpretation	55
5	Optic nerve head evaluation	27
Progression monitoring		
1	Differentiating glaucomatous changes from myopic changes	91
2	Progression analysis with different devices and programs	86
3	Visual field testing and interpretation	80
4	Role and reliability of OCT	56
5	Role of IOP and target IOP	28
Nonsurgical treatment		
1	Effectiveness of different medications	111
2	Effectiveness of SLT	97
3	Target IOP	43
4	Side effects of medication	23
5	Role of neuroprotection	22
Surgical treatment		
1	Choice of glaucoma surgery	179
2	Surgical risks	88
3	Effectiveness and safety of MIGS	31
4	Modifications in surgical technique	28
5	Antifibrotic treatment	18

*OCT* optical coherence tomography, *IOP* intraocular pressure, *SLT* selective laser trabeculoplasty, *MIGS* Minimally Invasive Glaucoma Surgery.

## Discussion

This survey helped us understand how many unresolved issues glaucoma specialists face in their daily clinical work in the diagnostics, progression monitoring, nonsurgical, and surgical treatment of glaucoma in myopic eyes. The survey highlights some major knowledge gaps among specialists in the field of glaucoma in myopic eyes. Our survey results provide a basis for defining research priorities for glaucoma research in myopic eyes. The three most frequent themes were differentiating glaucomatous changes from myopia, choice of glaucoma surgery, and role and reliability of OCT in myopic eyes.

The survey also formed the foundation for our Glaucoma and Myopia Project. Based on the survey responses, we formulated 87 umbrella questions that guide a comprehensive systematic literature review. The purpose of the ongoing literature review is to explore the evidence base that may provide answers to the participants’ questions. The subsequent steps involve summarising the evidence for the questions, highlighting gaps in the literature, and, where appropriate, developing consensus statements from the working group to provide clinical guidance in areas where evidence is limited [37].

Ten percent of the questions addressed how glaucomatous changes and progression can be better distinguished from myopia. The challenges of evaluating glaucomatous defects in myopic eyes have previously been discussed by Jiravarnsirikul et al. [21]. Regarding visual field testing, the respondents addressed difficulties in interpreting visual fields in myopic eyes because retinal myopic structural changes may lead to field defects. Related to these concerns, Lin et al have proposed a classification system for VF abnormalities in high myopia, including glaucoma-like defects observed in eyes without glaucoma [38].

The third most recurring theme in the survey was the role and reliability of OCT. Survey participants asked various questions about the ideal OCT parameters and biomarkers in myopic eyes. The participants also noted that one of the critical issues is the lack of a reliable normative database of myopic individuals for OCT. Normative databases of myopic eyes have been collected and studied, but not implemented in the OCT programs [39–44]. To support this effort, the Glaucoma/Myopia OCT Phenotyping Consortium was formed in 2020 with an aim to collate OCT datasets that consist of highly myopic patients without glaucoma, highly myopic patients with glaucoma, and non-highly myopic patients with early glaucoma [45].

For surgical treatment, the most frequent question themes were the choice of glaucoma surgery; balancing risk and effectiveness of surgical treatment in myopic eyes, and whether the presence of high myopia should change the surgical approach. Although our subsequent literature review found several publications on this topic [22–32], most available studies are retrospective, cross-sectional and/or non-comparative and do not provide sufficient evidence to fully answer these survey questions. Prospective and comparative surgical studies are needed to support clinical decision-making in the surgical treatment of glaucoma in myopic eyes. Regarding the risks of surgery, myopia is a known risk factor for hypotony after glaucoma surgery [46–49], but little is known about its prevention or treatment [25, 48].

The effectiveness of medications and of SLT was an area of enquiry for most participants in the nonsurgical treatment theme. After a subsequent systematic screen of the literature in PubMed and Embase on these topics, we found that no reports have been published on medical IOP reduction specifically for myopic eyes, although one randomised control trial is ongoing [33]. We found no publications on the effect of SLT in myopic eyes with glaucoma, although 5% of the survey questions addressed it. The safety of SLT appeared good in myopia in a study of 648 SLT treatments, with only one complication that affected vision [50]. For the future, we suggest nonsurgical treatment as a high-priority research topic in glaucoma in myopic eyes. For the purpose of the survey, we did not define myopia or high myopia. We recommend using the definitions by Glaucoma/Myopia OCT Phenotyping Consortium and International Myopia Institute in future studies. Both groups use both refractive error and axial length to define high myopia [45, 51].

A limitation of our study is its representativeness. The response rate was 24%, and while this figure seems like a low participation rate, it is typical for an online survey that is sent via email [52]. Considering the representation, our study reflects a European perspective, as 82% of participants reported working in Europe. Another limitation is that only 13% of participants estimated that they see more than 20 myopic glaucoma patients or myopic glaucoma suspects weekly. The low weekly caseload of myopic patients suggests that our participants may have limited experience in treating glaucoma in these eyes. In Asia, myopia clinics are already well-established, while in Europe there may be an unmet need for clinics focusing on myopic patients, which could help build clinical expertise and improve care for this growing patient population.

Although our survey generated a large number of free-text responses, categorising them allowed us to highlight priorities for future research on glaucoma in myopic eyes. Based on the nature of our study, we used a qualitative approach in our analysis, which should be noted when interpreting the survey results. Because the majority of the responses were in free text, we decided to use large language models to reduce direct human interpretation bias. Since the LLMs are also not fully transparent, we took an additional step in validating the results with human interpretation.

In the current study, we identified questions and clinical challenges for which there is a lack of knowledge among glaucoma specialists. The three most common themes in the diagnostics and progression monitoring categories were differentiating glaucoma from myopia and improving OCT and VF reliability and evaluations in the setting of myopia. The three most common themes in the treatment of glaucoma in myopic eyes were the choice of glaucoma surgery, effectiveness of different topical medications, and effectiveness of SLT. We propose prioritising future research into these topics.

## Summary

### What was known before

- Glaucoma diagnostics and management is challenging in eyes with myopia.

### What this study adds

- We propose prioritising future research into these topics: choice of glaucoma surgery, effectiveness of different topical medications and SLT in myopic eyes with glaucoma, differentiating glaucoma from myopia and improving OCT and VF reliability and evaluations in the setting of myopia.

## Data Availability

The datasets generated during and/or analysed during the current study are available from the corresponding author on reasonable request.
